# The Committee on Legislation

**Published:** 1897-08

**Authors:** 


### EDITORIAL.

The office of the Business Manager of THE JOURNAL is 311 and 312 Fitten Building.

The editors are not responsible for views expressed by contributors.

Articles for publication should reach this office not later than the 15th inst.

Address all communications, and make all remittances payable to THE ATLANTA MEDICAL AND SURGICAL JOURNAL, Atlanta, Ga.

Reprints of articles will be furnished, when desired, at a small cost. Requests for the same should always be made *on the manuscript.*

### THE COMMITTEE ON LEGISLATION.

At the late meeting of the State Medical Association in Macon resolutions were adopted providing for the appointment of a Committee on Legislation whose duty it should be to prepare for the next legislature certain bills of interest to the medical profession and to accomplish, if possible, their enactment into laws. This committee has been appointed, and we invite attention to a communication from the chairman on another page.

This is given us too late for extended comment now. Suffice it to say for the present that we are in thorough sympathy with the spirit of these resolutions, particularly that portion of them which contemplates some improvement in our present methods of selecting medical expert testimony. There is a crying need for reform here, as the court records in every county in Georgia can testify, and as recent events in this community have abundantly demonstrated. We also regard the proposition to insist upon higher qualifications preliminary to the study of medicine as eminently proper.

At another time we will have more to say.

